# Measurement of glomerular filtration rate reveals that subcapsular injection of shear‐thinning hyaluronic acid hydrogels does not impair kidney function in mice

**DOI:** 10.1002/jbm.a.37317

**Published:** 2021-09-30

**Authors:** Danielle E. Soranno, Lara Kirkbride‐Romeo, Daniel Han, Christopher Altmann, Christopher B. Rodell

**Affiliations:** ^1^ Department of Pediatrics University of Colorado Aurora Colorado USA; ^2^ Department of Bioengineering University of Colorado Aurora Colorado USA; ^3^ Department of Medicine University of Colorado Aurora Colorado USA; ^4^ Department of Urology Stanford University CA USA; ^5^ School of Biomedical Engineering, Science and Health SystemsScience and Health Systems Drexel University Philadelphia Pennsylvania USA

**Keywords:** biocompatibility, glomerular filtration rate, hyaluronic acid, injectable hydrogels, kidney function

## Abstract

The continued development of minimally invasive therapeutic implants, such as injectable hydrogels, necessitates the concurrent advancement of methods to best assess their biocompatibility via functional outcomes in vivo. Biomaterial implants have been studied to treat kidney disease; however, assessment of biocompatibility has been limited to biomarker and histological assessments. Techniques now exist to measure kidney function serially in vivo in murine studies via transcutaneous measurements of glomerular filtration rate (tGFR). In this study, adult male and female wild‐type BalbC mice underwent right unilateral nephrectomy. The remaining solitary left kidney was allowed 4 weeks to recover via compensatory hypertrophy, after which subcapsular injection of either saline or shear‐thinning hyaluronic acid hydrogel was performed. Serial tGFR measurements before and after treatment were used to assess the effect of hydrogel injection on kidney filtration. Urine and serum biomarkers of kidney function, and kidney histology were also quantified. Hydrogel injection did not affect kidney function, as assessed by tGFR. Results were in agreement with standard metrics of serum and urine biomarkers of injury as well as histological assessment of inflammation. The model developed provides a direct functional assessment of implant compatibility for the treatment of kidney disease and impact on kidney function.

## 1 INTRODUCTION

Injectable hydrogels have been widely investigated for biomaterial applications for their facile delivery of growth factors and cell‐based therapies.^1^, ^2^, ^3^, ^4^, ^5^, ^6^, ^7^, ^8^, ^9^ Numerous studies have been published investigating injectable hydrogel systems for the treatment of kidney disease in preclinical models. Various hydrogel systems have been utilized, including chitosan, collagen, polyethylene‐glycol, gelatin, and hyaluronic acid (HA)‐based gels to deliver cytokines, growth factors, and cell‐based therapies in models of acute and chronic kidney disease.^10^, ^11^, ^12^, ^13^, ^14^, ^15^, ^16^, ^17^, ^18^, ^19^, ^20^

As with any biomaterial application, the safety and biocompatibility of the system are of paramount importance for translational investigation. When utilized in the treatment of kidney disease, preclinical studies to‐date have based determinants of hydrogel biocompatibility on histological outcomes and biomarkers of kidney function. Histology has been used to determine the inflammatory response to the biomaterial. Serum biomarkers of kidney function, such as blood urea nitrogen (BUN) and serum creatinine (SCr) have been used to assess the effect of the hydrogel on kidney function. Kidneys filter BUN and creatinine, excreting these solutes into the urine, so a rise in either of these biomarkers can indicate a decline in kidney function. BUN and SCr are clinically relevant and commonly utilized, however, they have significant limitations when it comes to assessing kidney function. Both BUN and SCr are poor predictors of actual glomerular filtration rate (GFR) and their accuracy is dependent upon the assay utilized.^21^, ^22^ GFR measures the rate of plasma filtered by the kidneys per minute. In animal models with two kidneys, the GFR reflects the filtration of both kidneys combined. In a solitary kidney model, the GFR reflects the filtration rate of the solitary kidney. A decrease in GFR is used to define acute kidney injury and chronic kidney disease. While serum biomarkers such as BUN and SCr are commonly utilized to estimate changes in GFR, their ability to detect kidney injury is not sensitive.^23^, ^24^, ^25^ Measurements of GFR remain the gold standard for assessment of kidney function, however, such measurements are timely, invasive, and costly.^26^ The National Institute of Diabetes and Digestive and Kidney Disease (NIDDK) published recommendations on improving the translatability of animal models of kidney disease and highlighted the importance of accurate measurement of kidney function.^27^ There is now a commercially available method to measure GFR in rats and mice via minimally invasive transcutaneous measurement.^28^, ^29^

We have previously demonstrated the ability to measure the GFR serially for 1 year in a murine model of bilateral ischemia–reperfusion acute kidney injury.^30^ Specifically germane to unilateral hydrogel therapy, we have measured the GFR of one kidney of interest by performing a contralateral nephrectomy and allowing the kidney of interest to recover for 4 weeks. Following the unilateral contralateral nephrectomy, the isolated kidney of interest begins to hyperfilter in order to compensate for the increased workload. We have shown that 4 weeks of recovery provides adequate time for the solitary kidney to provide normal GFR following ischemia–reperfusion injury.^31^

To‐date, measured GFR has not been included in the assessment of the biocompatibility of biomaterial applications to treat kidney disease. We have previously utilized our injectable HA hydrogels to mitigate kidney disease progression in both acute and chronic kidney disease models in mice.^18^, ^19^ We have demonstrated that HA does not negatively affect either biomarkers of kidney function (BUN and SCr) nor renal histology. Indeed, HA hydrogels have mitigated the progression of kidney fibrosis in both ischemic and obstructive murine models of kidney disease. The purpose of this study was to demonstrate a method by which kidney function may be directly measured after treatment with our injectable HA hydrogel system in preclinical murine models. We hypothesized that delivery of HA hydrogel under the kidney capsule would not negatively impact measured GFR. Measured functional outcomes can be included in the preclinical biocompatibility assessment of biomaterials used to treat kidney disease, thus improving the translatability and clinical feasibility of local biomaterials in the treatment of kidney disease.

## 2 MATERIALS AND METHODS

### 2.1 Animals

All animal procedures and care conformed to the National Institutes of Health Guide for the Care and Use of Laboratory Animals and were approved by the Institutional Animal Care and Use Committee at the University of Colorado. Adult (6–7 week old) wild‐type BalbC male and female mice were acquired (Jackson Laboratories, Bar Harbor, ME) and were housed in standard conditions with *po* ad lib access to water and standard chow.

### 2.2 Measurement of transcutaneous glomerular filtration rate

Serial transcutaneous glomerular filtration rates (tGFR) were measured as previously described.^31^ Briefly, FITC‐sinistrin was administered intravenously via retro‐orbital injection and the renal clearance was measured for 65 min per manufacturer instructions (MediBeacon GMBH, Manheim, Germany). Measurements of tGFR are normalized for body weight. Pre‐nephrectomy, measured tGFR represents the function of both kidneys. Measurements obtained post‐unilateral nephrectomy reflect the kidney function of the remaining solitary kidney.

### 2.3 Unilateral nephrectomy

On study Day 0, a right unilateral nephrectomy was performed as previously described.^31^ The animals were given 4 weeks to recover postoperatively in order to allow for compensatory hypertrophy of the remaining left solitary kidney.

### 2.4 Delivery of injectable hydrogel under the left kidney capsule

On study Day 28, the animals received one of the three following treatments: (1) no treatment (Control), (2) 15 μl of normal saline injected under the left kidney capsule (Saline), or (3) 15 μl HA hydrogel injected under the left kidney capsule (HA). HA hydrogels were formulated and subcapsular injections were performed as previously described.^18^, ^19^ In brief, HA (74 kDa) was pendently modified by either adamantane (Ad‐HA) or β‐cyclodextrin (CD‐HA), with each component having an average of 20%–25% of disaccharides modified by the pendant group.^32^ For optical imaging studies, Ad‐HA was further labeled by the near‐IR dye Cy7.5 via methacrylation of CD‐HA and subsequent Michael‐addition with a fluorophore‐terminated peptide containing a cysteine residue (GCKKG‐Cy7.5).^33^ Hydrogels were prepared by dissolution of the polymers at 3.5 wt % in sterile saline and mixing to afford a shear‐thinning and injectable hydrogel.

### 2.5 Serial in vivo optical imaging

Hydrogel localization under the kidney capsule was verified by dorsal and lateral images, while hydrogel quantification was performed on the lateral images. Hydrogel degradation was tracked via optical imaging utilizing the Pearl (LI‐COR) small animal imaging machine, as previously described.^16^, ^18^ Signal intensity was measured in photons/pixel/second and normalized for maximum intensity per animal. Animals treated with HA hydrogel underwent serial optical imaging daily for 3 days, then again on Day 33 and prior to sacrifice on study Day 35.

### 2.6 Biomarker measurement

Serum was collected via retro‐orbital blood draw at baseline and on study Day 1 (1 day after unilateral nephrectomy), Day 27 (pretreatment), Day 29 (posttreatment), and collected at sacrifice via cardiac puncture on Day 35. Serum was processed as previously described.^31^ Urine, when available was collected noninvasively at the same time points and via bladder puncture at sacrifice on Day 35. Assays for serum BUN (BioAssay Systems QuantiChrom™ Urea Assay Kit), enzymatic creatinine (Pointe Scientific Creatinine Enzymatic Reagent Kit), cystatin C (R&D Systems, Minneapolis, MN), and urine kidney injury molecule‐1 (KIM‐1) (R&D Systems, Minneapolis, MN) and neutrophil gelatinase‐associated lipocalin (NGAL) (R&D Systems, Minneapolis, MN) were performed per manufacturer's instructions.

### 2.7 Histological quantification of kidney fibrosis

Kidney tissue was formalin‐fixed, paraffin embedded, and sectioned (4 μm thickness) for staining. Kidney fibrosis was assessed by staining and quantifying via picrosirius red as previously described^31^; hydroxyproline content was measured as previously described.^31^ Only the left treated kidneys were analyzed; the right nephrectomized kidneys were flash frozen.

### 2.8 Statistics

Inference of the means assuming normal distribution was used to determine the number of mice required per cohort in order to detect a 15% change in measured tGFR with 85% power, *p* < .05; each experimental cohort contains *n* = 7 males and *n* = 7 females. *T*‐tests were utilized to compare measures between two groups, assuming Gaussian distribution and using Welch's correction. Paired *T*‐tests were utilized to compare the change in measured tGFR within the same group over time. ANOVA was utilized for comparison amongst three groups with Tukey post hoc analysis. Males and females were analyzed separately to ensure there was no difference with regards to sex as a biologic variable.^34^ Data represented in figures show mean ± SEM.

## 3 RESULTS

**FIGURE 1:**
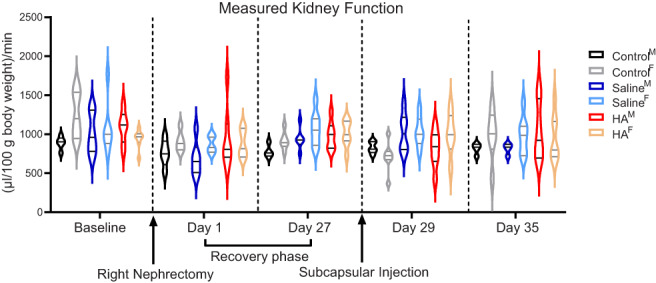
Violin plot of measured transcutaneous glomerular filtration rate (tGFR), normalized for weight, over time. TGFR was assessed serially at Baseline, 1 day status‐post right nephrectomy (Day 1), 4 weeks status‐post right nephrectomy (Day 27), 1 day after treatment with either Saline or hyaluronic acid hydrogel (Day 29) and at sacrifice (Day 35). There was no statistically significant difference in tGFR amongst the cohorts at any individual time point. M, males; F, females

There was no difference between serial tGFR measurements normalized for weight amongst the groups at baseline (pre‐unilateral nephrectomy), on Day 1 (post‐unilateral nephrectomy), on Day 27 (pretreatment), Day 29 (posttreatment), or at sacrifice on Day 35 (Figure 1). Furthermore, there was no difference in normalized tGFR between the male and female cohorts.

**FIGURE 2:**
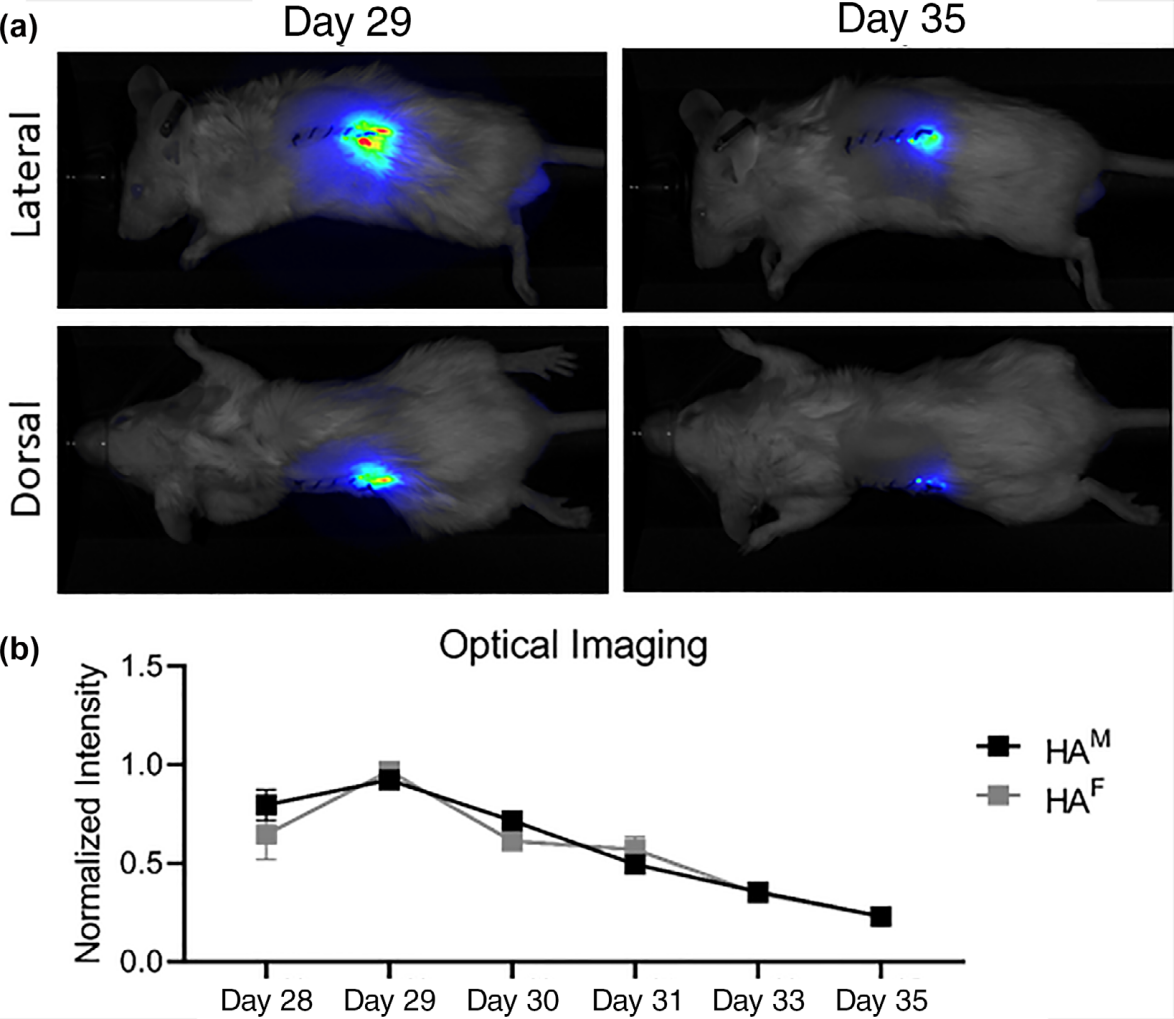
Hyaluronic acid (HA) hydrogel localization and quantification over time. Optical imaging was performed serially after HA injection under the left kidney capsule. (A) Representative lateral and dorsal images of a male mouse on the day after injection (Day 29) and 1 week later prior to sacrifice (Day 35). (B) Quantification of HA hydrogel demonstrated no difference between males and females in normalized signal intensity over time (*n* = 7). M, males; F, females

Cohorts treated with HA hydrogel under the left kidney capsule on Day 28 underwent serial optical imaging until sacrifice on Day 35. Figure 2 shows the sustained degradation of HA after injection, with no difference between male and female cohorts.

**FIGURE 3:**
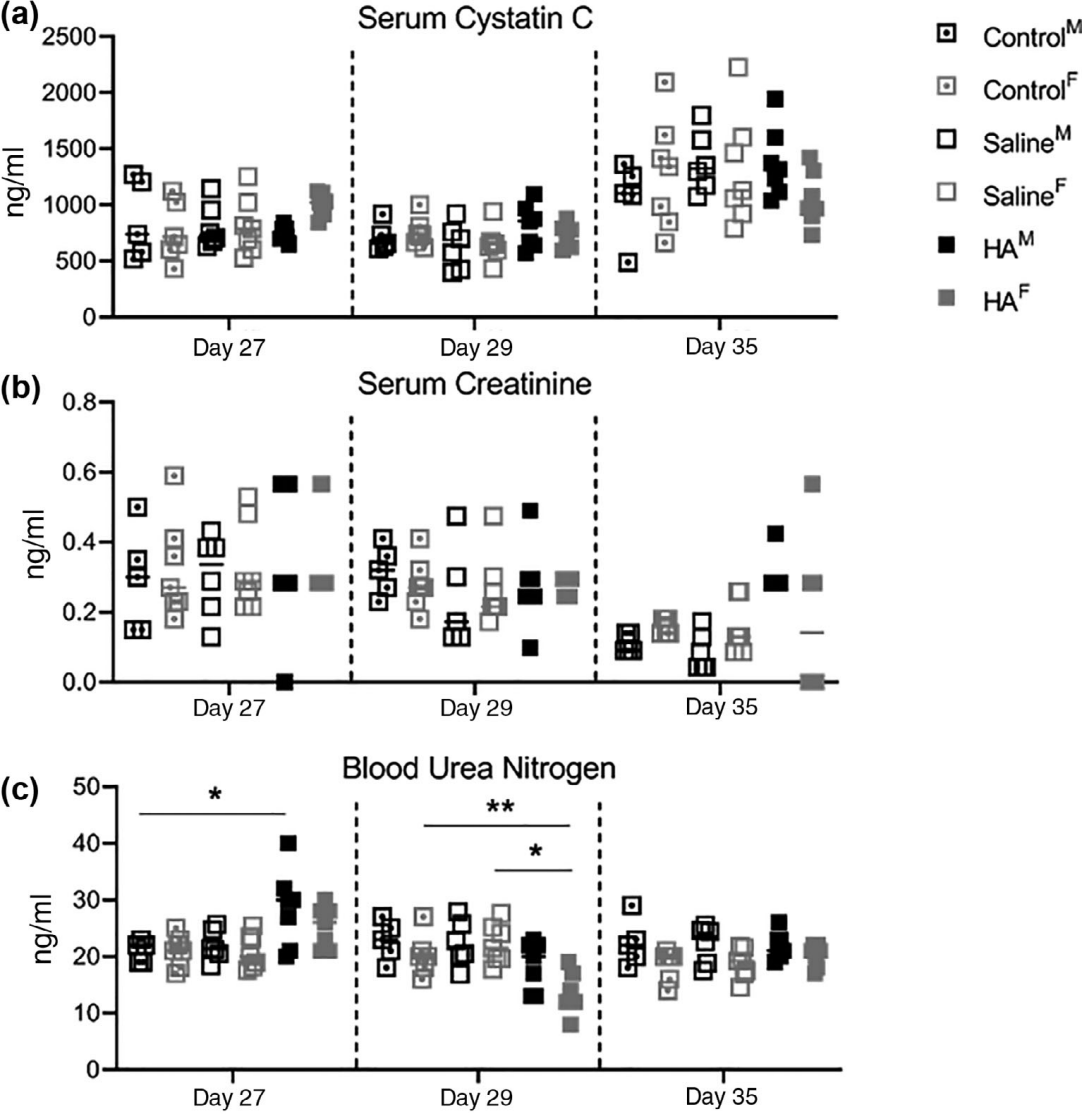
Biomarkers of kidney function over time. (A) Serum cystatin C, (B) serum creatinine, and (C) blood urea nitrogen were measured 1 month after the right nephrectomy and pretreatment (Day 27), 1 day after treatment (Day 29) and at sacrifice 1 week after treatment (Day 35). Blood urea nitrogen was higher in males that had not yet received HA on Day 27 and lower in females 1 day after treatment with HA compared to female control and saline groups (*n* = 7). *p < .05, **p < .01. M, males; F, females

One day after the right nephrectomy (Day 1), there were no statistically significant differences in serum biomarkers of kidney function (Cystatin C, SCr, and BUN) (Figure S1). There was no difference in cystatin C (Figure 3A) or SCr (Figure 3B) prior to treatment (Day 27), 1 day after treatment (Day 28), or 1 week after treatment (Day 35). Male mice that had not yet received HA hydrogel demonstrated a slight increase in BUN on the day prior to treatment (Day 27), but the elevation did not persist posttreatment (Figure 3C). One day after HA treatment (Day 29), female mice that had received HA demonstrated lower BUN measurements compared to both the female Control and Saline groups. There was no difference in BUN amongst any of the groups prior to sacrifice on Day 35. In our experience, normative biomarker values in young adult mice are: BUN 10–20 mg/dl; SCr 0.15–0.3 mg/dl and serum cystatin C < 2000 ng/ml (<0.2 mg/dl).

**FIGURE 4:**
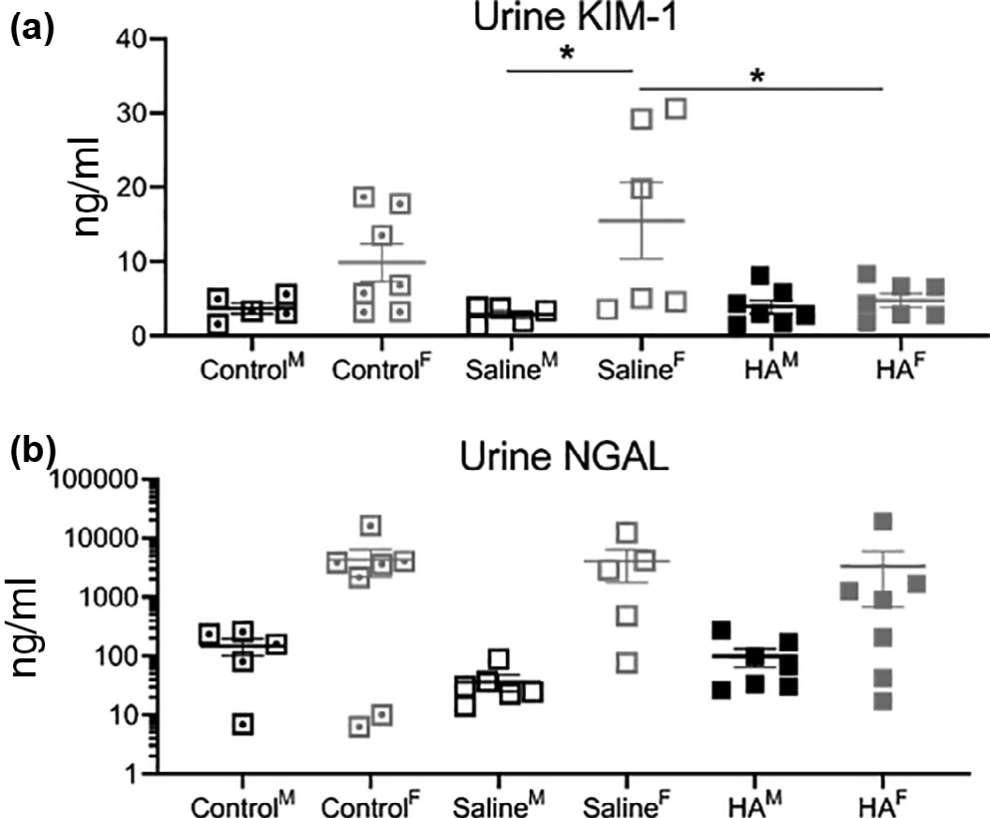
Urine biomarkers of kidney injury at sacrifice. (A) Urine kidney injury molecule‐1 (KIM‐1) and (B) urine gelatinase‐associated lipocalin (NGAL) were obtained via bladder puncture at sacrifice. Females treated with Saline under the kidney capsule had elevated KIM‐1 levels compared to either males that had received Saline or females that had received hyaluronic acid (*n* = 7). *p < .05. M, males; F, females

Urine was collected serially when available. Earlier time points were underpowered, however, the available data are shown in Figure S2. At sacrifice on Day 35 urine KIM‐1 levels were increased in Saline^F^ compared to both Saline^M^ and HA^F^ groups (Figure 4A). There was no difference in urine NGAL levels amongst the cohorts (Figure 4B).

**FIGURE 5:**
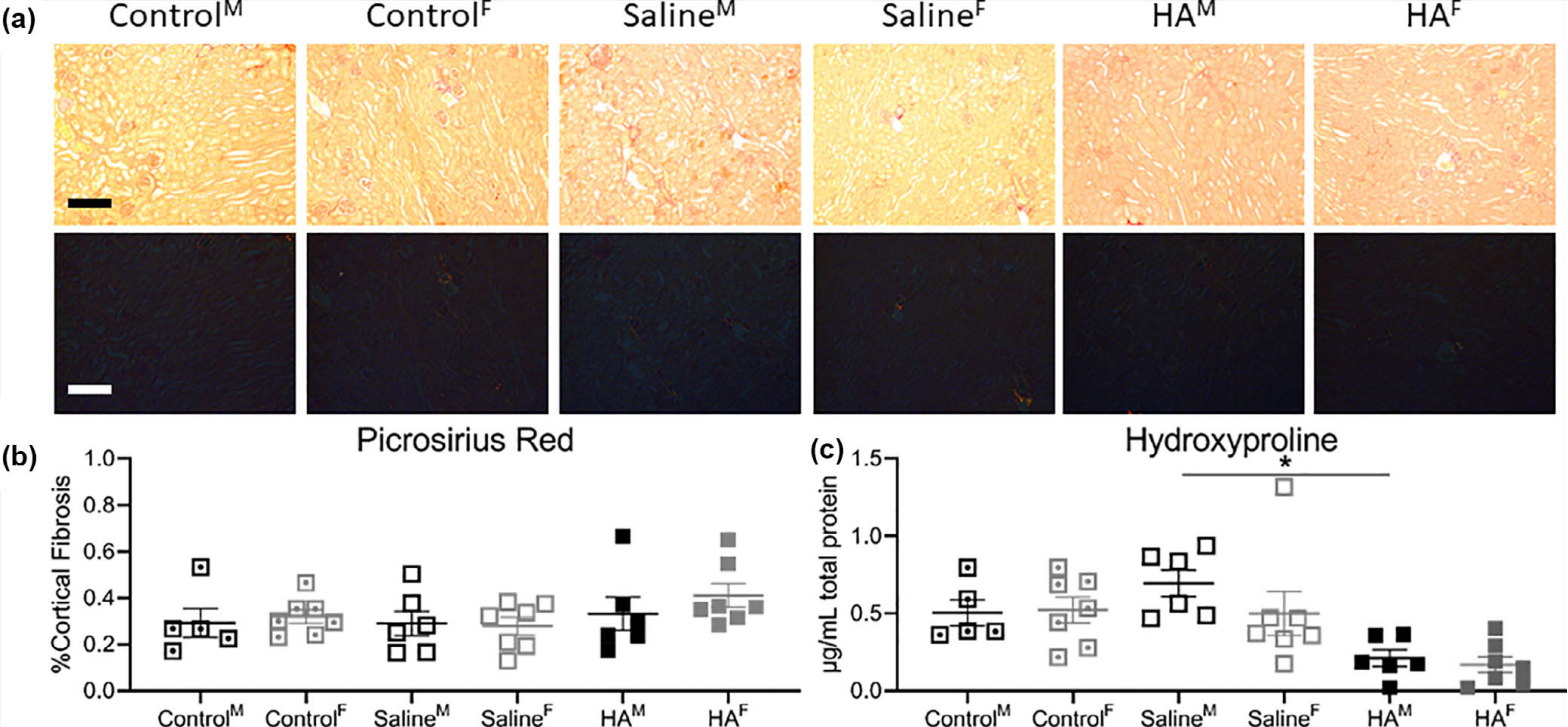
Kidney fibrosis at sacrifice. (A) Light (top panel) and polarized (bottom panel) picrosirius red images, (B) picrosirius red quantification, (C) hydroxyproline content all demonstrate that hyaluronic acid hydrogel treatment does not increase kidney fibrosis. Magnification, ×200. Scale bar = 10 μM. *n* = 7, *p* < .05. M, males; F, females

Figure 5 shows the histological outcomes of fibrosis after sacrifice on Day 35. Overall, kidney fibrosis was low in all groups and across both markers of fibrosis (picrosirius red and hydroxyproline content). There was no difference amongst the cohorts in the quantification of picrosirius red polarized images (Figure 5B), nor hydroxyproline content (Figure 5C).

## 4 DISCUSSION

We have previously utilized our injectable HA hydrogel to provide unilateral therapy in murine models of acute and chronic kidney disease and have shown histological improvement in groups treated with HA hydrogel alone.^18^, ^19^ These effects may be due in part to the cytoprotective, antifibrotic, and immunomodulatory effects of HA itself, observed by ourselves and others.^35^, ^36^ Serum biomarkers of kidney function have also indicated that HA hydrogels are biocompatible and do not impair renal function. Biomarkers, however, only *estimate* kidney function. To‐date, functional *measurements* of kidney filtration have not been included in the biocompatibility assessment of biomaterials designed for in situ treatment of kidney disease.

In order to isolate the treated (left) kidney, we first performed a unilateral right nephrectomy. We have previously demonstrated that 4 weeks is sufficient time for the contralateral kidney to compensate in a model of unilateral renal ischemia–reperfusion injury.^31^ Having isolated the left kidney, we then measured tGFR pre‐ and post‐HA hydrogel treatment. Our data demonstrate that delivery of HA under the kidney capsule does not adversely affect measured kidney function. Serum estimates of kidney function and urine biomarkers of kidney injury were similarly reassuring. Indeed, BUN—which is a more sensitive and less specific biomarker of kidney injury than either SCr or cystatin C, was lower in female HA cohorts compared to the female saline and unmanipulated controls. This may be secondary to local effects of saline which can elicit metabolic acidosis and acute kidney injury.^37^ Other groups have studied renal outcomes after injecting the hydrogel therapy directly into the renal parenchyma, while we utilize a subcapsular approach in order to avoid causing any direct damage to functioning nephrons. Such variations in injection technique may impact how the hydrogel affects kidney function and warrant further study.

Our optical imaging aligns with our prior studies in which we delivered the HA hydrogel in both obstructive^18^ and ischemia–reperfusion^19^ models of kidney injury, suggesting that subcapsular HA hydrogel degradation is not affected by acute or chronic kidney injury.

Our study has numerous strengths. The addition of tGFR measurements to standard serum and urine biomarker and histology assessment aligns with the NIDDK's recommendation on overcoming barriers in translational research for kidney disease.^27^ Likewise, by including both male and female mice and presenting the data unpooled by sex, our study aligns with current recommendations to improve scientific rigor and transparency in translational research.^34^ Sex hormones have been demonstrated to effect outcomes in ischemia–reperfusion acute kidney injury, nephrotoxic‐mediated kidney injury, and the development of chronic kidney disease.^11^, ^38^, ^39^, ^40^ Overall, our data suggest that sex does not affect renal outcomes after HA delivery under the kidney capsule in healthy mice.

## 5 CONCLUSION

In conclusion, we have described a quantitative way to measure the effect that injectable hydrogels have on GFR in murine models. Unilateral treatment of hydrogel to one kidney can be assessed after performing a contralateral unilateral nephrectomy. Assessment of measured kidney function is an important marker of biocompatibility for biomaterial systems used to treat kidney disease and should be incorporated into the assessment of preclinical treatment models in addition to standard biomarkers and histology. Direct in vivo assessments of kidney function can ensure safety and efficacy beyond that of biomarkers and histological outcome.

## CONFLICT OF INTEREST

The authors declare no potential conflict of interest.

## Supporting information


**Figure S1** Biomarkers of kidney function 1 day after right nephrectomy. (A) Serum cystatin C, (B) Serum creatinine, and (C) Blood urea nitrogen were measured 1 day after the right nephrectomy (Day 1). There was no significant difference amongst the future treatment groups in the three serum biomarkers. (n = 7) * *p* < .05, ** *p* < .01. M, males; F, females.

**Figure S2** Urine biomarkers of kidney injury over time. (A) Urine KIM‐1 and (B) Urine NGAL over time. Serial urine collections were limited by availability. M, males; F, females.

## Data Availability

The raw/processed data required to reproduce these findings can be obtained by emailing the corresponding author.
