# Supplementary figures and images for: Measurement of glomerular filtration rate reveals that subcapsular injection of shear‐thinning hyaluronic acid hydrogels does not impair kidney function in mice
Source: J Biomed Mater Res A. 2021 Sep 30;110(3):652–8. doi: 10.1002/jbm.a.37317 (PMC9292789; doi:10.1002/jbm.a.37317)

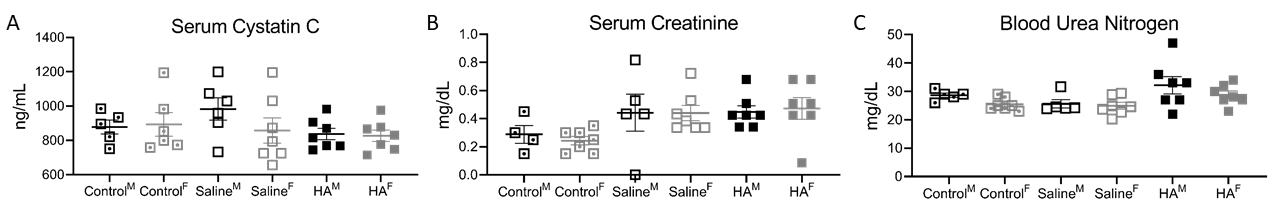

Supplement: Supplementary file 1 — Figure S1 Biomarkers of kidney function 1 day after right nephrectomy. (A) Serum cystatin C, (B) Serum creatinine, and (C) Blood urea nitrogen were measured 1 day after the right nephrectomy (Day 1). There was no significant difference amongst the future treatment groups in the three serum biomarkers. (n = 7) * p < .05, ** p < .01. M, males; F, females. [file JBM-110-652-s001.tif]

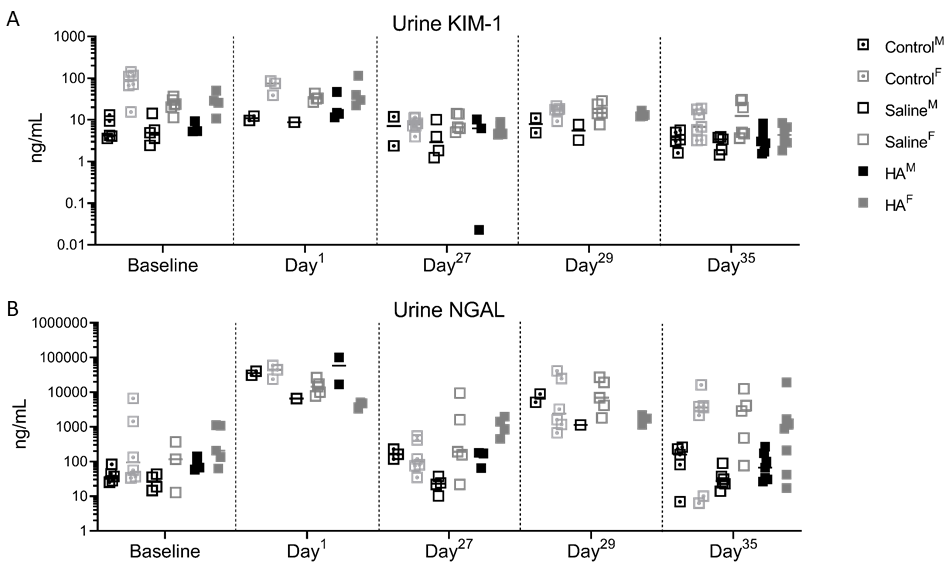

Supplement: Supplementary file 2 — Figure S2 Urine biomarkers of kidney injury over time. (A) Urine KIM‐1 and (B) Urine NGAL over time. Serial urine collections were limited by availability. M, males; F, females. [file JBM-110-652-s002.tif]
